# Long-Term Efficacy of T3 Analogue Triac in Children and Adults With MCT8 Deficiency: A Real-Life Retrospective Cohort Study

**DOI:** 10.1210/clinem/dgab750

**Published:** 2021-10-22

**Authors:** Ferdy S van Geest, Stefan Groeneweg, Erica L T van den Akker, Iuliu Bacos, Diana Barca, Sjoerd A A van den Berg, Enrico Bertini, Doris Brunner, Nicola Brunetti-Pierri, Marco Cappa, Gerarda Cappuccio, Krishna Chatterjee, Alexander D Chesover, Peter Christian, Régis Coutant, Dana Craiu, Patricia Crock, Cheyenne Dewey, Alice Dica, Paul Dimitri, Rachana Dubey, Anina Enderli, Jan Fairchild, Jonathan Gallichan, Luigi R Garibaldi, Belinda George, Annette Hackenberg, Bianka Heinrich, Tony Huynh, Anna Kłosowska, Amy Lawson-Yuen, Michaela Linder-Lucht, Greta Lyons, Felipe Monti Lora, Carla Moran, Katalin E Müller, Laura Paone, Praveen G Paul, Michel Polak, Francesco Porta, Christina Reinauer, Yolanda B de Rijke, Rowen Seckold, Tuba Seven Menevşe, Peter Simm, Anna Simon, Marco Spada, Athanasia Stoupa, Lilla Szeifert, Davide Tonduti, Hans van Toor, Serap Turan, Joel Vanderniet, Monique de Waart, Ronald van der Wal, Adri van der Walt, Anne-Marie van Wermeskerken, Jolanta Wierzba, Federica Zibordi, Amnon Zung, Robin P Peeters, W Edward Visser

**Affiliations:** 1 Academic Center for Thyroid Diseases, Department of Internal Medicine, Erasmus Medical Center, 3015 GD Rotterdam, The Netherlands; 2 Division of Endocrinology, Department of Pediatrics, Erasmus MC-Sophia Children’s Hospital, University Medical Center, 3015 GD Rotterdam, The Netherlands; 3 Centrul Medical Dr. Bacos Cosma, Timisoara 307200, Romania; 4 Carol Davila University of Medicine, Department of Clinical Neurosciences, Paediatric Neurology Discipline II, Bucharest 050474, Romania; 5 Paediatric Neurology Clinic, Reference Center for Rare Paediatric Neurological Disorders, ENDO-ERN member, Alexandru Obregia Hospital, Bucharest 041914, Romania; 6 Diagnostic Laboratory for Endocrinology, Department of Internal Medicine, Erasmus Medical Center , 3015 GD Rotterdam, The Netherlands; 7 Department of Clinical chemistry, Erasmus Medical Center, 3015 GD Rotterdam, The Netherlands; 8 Unit of Neuromuscular and Neurodegenerative Disorders, Bambino Gesu’ Children’s Research Hospital IRCCS, 00165 Rome, Italy; 9 Gottfried Preyer’s Children Hospital, 1100 Vienna, Austria; 10 Department of Translational Medicine, Federico II University, 80131 Naples, Italy; 11 Telethon Institute of Genetics and Medicine, Pozzuoli, 80078 Naples, Italy; 12 Division of Endocrinology, Bambino Gesu’ Children’s Research Hospital IRCCS, 00165 Rome, Italy; 13 Wellcome Trust-Medical Research Council Institute of Metabolic Science, University of Cambridge, Cambridge CB2 0QQ, UK; 14 Division of Endocrinology, The Hospital for Sick Children and Department of Paediatrics, University of Toronto, Toronto, M5G 1X8, Canada; 15 East Kent Hospitals University NHS Foundation Trust, Ashford TN24 0LZ, UK; 16 Department of Pediatric Endocrinology and Diabetology, University Hospital, 49100 Angers, France; 17 John Hunter Children’s Hospital, New Lambton Heights, NSW 2305, Australia; 18 Hunter Medical Research Institute, University of Newcastle Kookaburra Circuit, New Lambton Heights, NSW 2305, Australia; 19 Genomics Institute Mary Bridge Children’s Hospital, MultiCare Health System Tacoma, WA 98403, USA; 20 Sheffield Children’s NHS Foundation Trust, Sheffield Hallam University and University of Sheffield, Sheffield, S10 2TH, UK; 21 Medanta Superspeciality Hospital, Indore 800020, India; 22 Department of Neuropediatrics, University Children’s Hospital Zurich, Steinwiesstrasse 75, 8032 Zürich, Switzerland; 23 Neurology Department, Children’s Hospital, St. Gallen, 9000, Switzerland; 24 Department of Diabetes and Endocrinology, Women’s and Children’s Hospital, North Adelaide 5066 SouthAustralia; 25 Plymouth Hospitals NHS Trust, Plymouth, PL6 8DH,UK; 26 UPMC Children’s Hospital of Pittsburgh, Pittsburgh, PA 15224, USA; 27 Department of Endocrinology, St. John’s Medical College Hospital, Bengaluru 560034, India; 28 Department of Endocrinology & Diabetes, Queensland Children’s Hospital, South Brisbane Queensland 4101, Australia; 29 Department of Chemical Pathology, Mater Pathology, South Brisbane, Queensland 4101, Australia; 30 Faculty of Medicine, University of Queensland, Brisbane, Queensland 4072, Australia; 31 Department of Pediatrics, Hematology and Oncology, Medical University of Gdańsk, 80-210 Gdańsk, Poland; 32 Division of Neuropediatrics and Muscular Disorders, Department of Pediatrics and Adolescent Medicine, University Hospital Freiburg, 79106 Freiburg, Germany; 33 Pediatric Endocrinology Group, Santa Catarina Hospital, São Paulo, 01310-000, Brazil; 34 Heim Pal National Institute of Pediatrics, Budapest, 1089, Hungary; 35 Institute of Translational Medicine, University of Pécs, Pécs, 7622, Hungary; 36 Department of Paediatrics, Christian Medical College, Vellore 632004, India; 37 Paediatric Endocrinology, Diabetology and Gynaecology Department, Necker Children’s University Hospital, Imagine Institute, Université de Paris, Paris 75015, France; 38 Department of Paediatrics, AOU Città della Salute e della Scienza di Torino, University of Torino, Torino 10126,Italy; 39 Department of General Pediatrics, Neonatology and Pediatric Cardiology, University Children’s Hospital, Medical Faculty, Duesseldorf 40225, Germany; 40 Marmara University School of Medicine Department of Pediatric Endocrinology, Istanbul 34854, Turkey; 41 Royal Children’s Hospital/University of Melbourne, Parkville 3052,Australia; 42 1st Department of Pediatrics, Semmelweis University, Budapest, 1083, Hungary; 43 Child Neurology Unit - C.O.A.L.A. (Center for Diagnosis and Treatment of Leukodystrophies), V. Buzzi Children’s Hospital, Milano 20154, Italy; 44 Private Paediatric Neurology Practice of Dr A van der Walt, Durbanville, South Africa; 45 Department of Paediatrics, Flevoziekenhuis, 1315 RA, Almere, The Netherlands; 46 Department of Internal and Pediatric Nursing, Institute of Nursing and Midwifery, Medical University of Gdańsk, 80-210 Gdańsk, Poland; 47 Child Neurology Unit, Fondazione IRCCS, Istituto Neurologico Carlo Besta, Milan 20133, Italy; 48 Pediatric Endocrinology Unit, Kaplan Medical Center, University of Jerusalem, Rehovot 76100, Israel

**Keywords:** MCT8 deficiency, Allan-Herndon-Dudley syndrome, AHDS, T3 analogue, thyromimetic drug

## Abstract

**Context:**

Patients with mutations in thyroid hormone transporter MCT8 have developmental delay and chronic thyrotoxicosis associated with being underweight and having cardiovascular dysfunction.

**Objective:**

Our previous trial showed improvement of key clinical and biochemical features during 1-year treatment with the T3 analogue Triac, but long-term follow-up data are needed.

**Methods:**

In this real-life retrospective cohort study, we investigated the efficacy of Triac in MCT8-deficient patients in 33 sites. The primary endpoint was change in serum T3 concentrations from baseline to last available measurement. Secondary endpoints were changes in other thyroid parameters, anthropometric parameters, heart rate, and biochemical markers of thyroid hormone action.

**Results:**

From October 15, 2014 to January 1, 2021, 67 patients (median baseline age 4.6 years; range, 0.5-66) were treated up to 6 years (median 2.2 years; range, 0.2-6.2). Mean T3 concentrations decreased from 4.58 (SD 1.11) to 1.66 (0.69) nmol/L (mean decrease 2.92 nmol/L; 95% CI, 2.61-3.23; *P* < 0.0001; target 1.4-2.5 nmol/L). Body-weight-for-age exceeded that of untreated historical controls (mean difference 0.72 SD; 95% CI, 0.36-1.09; *P* = 0.0002). Heart-rate-for-age decreased (mean difference 0.64 SD; 95% CI, 0.29-0.98; *P* = 0.0005). SHBG concentrations decreased from 245 (99) to 209 (92) nmol/L (mean decrease 36 nmol/L; 95% CI, 16-57; *P* = 0.0008). Mean creatinine concentrations increased from 32 (11) to 39 (13) µmol/L (mean increase 7 µmol/L; 95% CI, 6-9; *P* < 0.0001). Mean creatine kinase concentrations did not significantly change. No drug-related severe adverse events were reported.

**Conclusions:**

Key features were sustainably alleviated in patients with MCT8 deficiency across all ages, highlighting the real-life potential of Triac for MCT8 deficiency.

Thyroid hormone action is crucial for metabolic and developmental processes. Intracellular bioavailability of thyroid hormone is governed by plasma membrane transporters (1). Monocarboxylate transporter 8 (MCT8) is a specific thyroid hormone transporter that is crucial for transport of triiodothyronine (T3) and thyroxine (T4) in several tissues, including the brain (2). MCT8 deficiency (also known as *Allan-Herndon-Dudley Syndrome* or AHDS), caused by mutations in the MCT8-encoding gene *SLC16A2* on chromosome Xq13.2, is a rare disorder consisting of severe intellectual and motor disability and abnormal thyroid function tests (3-5). The neurocognitive phenotype, comprising severe developmental delay, dystonia, and central hypotonia is caused by defective MCT8, which impairs thyroid hormone entry into the brain (6, 7). In MCT8-independent tissues, the elevated serum T3 concentrations cause chronic thyrotoxicosis with negative clinical sequelae that include being underweight and having cardiovascular dysfunction (8). Together, these disease characteristics result in substantial morbidity throughout life and predispose to high mortality in childhood (8).

A therapeutic strategy involves the application of a thyroid hormone analogue that can enter cells independent of MCT8 and stimulate the endogenous thyroid hormone receptor. Preclinical studies showed that the T3 analogue Triac (3,3′,5-tri-iodothyroacetic acid; also known as *tiratricol*), whose cellular entry is not dependent on MCT8, fully rescued the brain phenotype in animal models of MCT8 deficiency when administered directly after birth (1, 9, 10). The ongoing Triac Trial II, a phase IIb clinical trial (NCT02396459) in young infants with MCT8 deficiency, will evaluate the effects of Triac on neurodevelopment once treatment is initiated early in life.

In a previous trial, in which pediatric and adult patients with MCT8 deficiency were treated with Triac for 12 months, we showed that key clinical and biochemical features due to peripheral thyrotoxicosis were ameliorated (11). The evaluation of therapies for rare disorders faces many challenges, including the uniform systematic collection of long-term data. Also, it is relevant to determine whether the efficacy of a treatment demonstrated in a clinical trial (typically with a limited number of patients intrinsic to the low prevalence) is applicable in a real-life setting. Such long-term follow-up data of patients with MCT8 deficiency treated with Triac are currently lacking, particularly in young patients.

In a joint international effort, we here retrospectively report long-term (up to 6 years) real-life follow-up data on children and adults with MCT8 deficiency treated with Triac.

## Methods

### Study Design and Participants

In this international pragmatic real-life cohort, data were collected retrospectively in patients with MCT8 deficiency, within our consortium of 33 hospitals in 18 countries, who have been treated with the T3 analogue Triac on an off-label use basis. Eligible subjects were those with MCT8 deficiency (confirmed by the presence of a pathogenic mutation in the *SLC16A2* gene), irrespective of age or comorbidities, who were treated with Triac on an off-label use basis following our previously established dose escalation protocol (11), and had their biochemical parameters measured in the central laboratory of the Erasmus MC, The Netherlands. This approach allowed for a uniform strategy of Triac dosing and monitoring of effects.

The cohort consisted of (1) patients who finished the Triac Trial I protocol and continued on Triac treatment on an off-label use basis; and (2) additional patients who were identified after the recruitment phase of Triac Trial I or could not be enrolled in the Triac Trial I due to the absence of study sites in their country. For patients who had been enrolled in the Triac Trial I, data from the assessments at baseline and after 1 year of Triac treatment have been re-utilized and extended by novel long-term follow-up data.

The parents or guardians of all patients provided consent for off-label use of Triac to their caregiving physician. Under off-label use and with retrospective collection of data that are part of routine clinical care from medical files, no institutional board approval was needed in the majority (30) of hub centers. Institutional board approval for off-label use of Triac was required and granted at the hub centers in Indore (India), Tacoma (USA) and Gdańsk (Poland). Data were collected prospectively during Triac Trial I, for which institutional board approval was granted at all study sites (11).

### Procedures

All patients discontinued antithyroid drugs, levothyroxine, or other thyroid hormone analogs (if applicable, eg, diiodothyropropionic acid [DITPA]) before treatment with Triac was initiated. After a washout period of 2 weeks, baseline measurements were recorded. All patients received Triac treatment (Téatrois or Emcitate tablets [350 µg], taken enterally; Rare Thyroid Therapeutics, Stockholm, Sweden) with individualized dose escalation based on the predefined dose escalation protocol of the Triac Trial I as guidance (11). The daily starting dose was based on body weight (175 µg Triac < 10 kg; 350 µg Triac ≥ 10 kg). The daily dose was increased with increments of 175 µg or 350 µg with a goal of attaining serum total T3 concentrations within the target range of 1.4 to 2.5 nmol/L. Guided by the exploratory neurodevelopmental outcomes in Triac Trial I (11), serum T3 concentrations < 1.4 nmol/L were allowed in children under 2.5 years of age in the absence of dose-limiting toxicities, leaving the possibility to administer higher Triac dosages, possibly increasing the potential for neurocognitive benefits.

Patients were assessed for study outcomes and drug-related adverse events at baseline and during follow-up visits. Guidance on Triac dosing was provided by Erasmus MC based on thyroid function tests (see Supplementary Methods (12)), which together with other blood components were measured in a central laboratory (Erasmus MC, Rotterdam, The Netherlands) according to routine procedures.

### Outcomes

The primary prespecified endpoint of this study was the change in serum T3 concentrations between baseline and last available measurement. The prespecified secondary endpoints were the change between baseline and last available body weight and body height (expressed as kg and cm, or weight-for-age and weight-for-height and height-for-age Z-scores, respectively, or as relative differences to the natural history reference; see Supplementary Methods (12)); heart rate (expressed as beats per minute, or heart-rate-for-age Z-score); serum thyroid-stimulating hormone (TSH; thyrotropin), free and total T4 concentrations; established biochemical markers that reflect thyroid hormone action in the liver (sex hormone-binding globulin [SHBG]), in the kidney (creatinine) and in muscle (creatine kinase [CK]); and Tanner stage of sexual maturation in patients who were aged between 8 and 18 years during treatment.

### Safety

The occurrence of (severe) adverse events related to Triac was actively monitored during treatment (see Supplementary Methods (12)).

### Statistical Analyses

Analysis on the primary outcome was based on the full analysis dataset, which included all patients who received at least 1 dose of Triac and who had at least 1 follow-up visit after the baseline assessment. Analyses on all prespecified secondary outcomes were done in all subjects who had a treatment duration that exceeded the mean time of dose escalation (defined as time from baseline measurement to the first follow-up visit on optimal dose), to ensure that treatment effects on secondary outcomes could be reliably assessed. Complementary analyses of secondary outcomes were performed on the full analysis dataset.

For all endpoints, *P* values and 95% CIs were calculated for the mean change between baseline and last available follow-up visit using paired *t* tests. Serum TSH and CK concentrations were first log-transformed to normalize the distribution. Complementary analyses after stratification by treatment duration (< 1 year, from 1 to 3 years, from 3 to 5 years and ≥ 5 years) were done using 1-way analysis of variance (ANOVA).

In longitudinal within-subject analyses of the primary and secondary endpoints, performed in all subjects who were treated for at least 2 years, baseline data were compared with those obtained after 1 year (±4 months) of treatment and at the last available follow-up visit within the same subject, using repeated-measurement ANOVA.

Missing data can mainly be attributed to the poor clinical condition of patients (including restricted availability of serum); common manifestations of MCT8 deficiency, such as scoliosis and dystonic posturing that hamper investigations for which patients needed proper positioning; and patients receiving clinical care outside the designated hub centers. With the assumption that omission of data occurred randomly, and given the broad age range of the participants and small group size, pairwise deletion was used to adjust when the baseline data point was missing; for missing data that were captured throughout the treatment period, the last available measurement was used (if available).

All statistical analyses were performed using GraphPad Prism version 5 (GraphPad Software Inc., San Diego, California, USA). Two-sided *P* values of less than 0.05 were considered to denote statistical significance.

### Role of the Funding Source

The funders of the study had no role in study design, data collection, data analysis, data interpretation, or writing of the report. The corresponding author had full access to all the data in the study and had final responsibility for the decision to submit for publication.

## Results

Between October 15, 2014, and January 1, 2020, 67 patients from 62 different families (with 46 different MCT8 mutations) had received Triac and were eligible for inclusion in this study. Among them, 27 patients had been enrolled in Triac Trial I and continued Triac treatment on an off-label use basis after completion of the trial protocol. Another 40 patients had been identified after the recruitment phase of Triac Trial I (n = 28) or could not be enrolled in the Triac Trial I due to the absence of study sites in their country (n = 12) (Supplementary Figure 1 (12)). Patients were treated in 33 different sites in 18 countries: 19 sites were located in Europe, 4 in the Americas, 5 in Asia, 4 in Oceania and 1 in Africa (Supplementary Figure 2 (12)).

At baseline, the median age was 4.6 years, with a range of 0.5 to 66 years. Of the 67 patients, 63 (94%) were younger than 18 years of age and 23 (34%) were younger than 2.5 years (Table 1 and Supplementary Figure 3A (12)). The median treatment duration was 2.2 years (range, 0.2-6.2 years). Twelve (18%) of the 67 patients were treated for a period of more than 5 years and 26 (39%) of 67 patients were treated for a period of 2 to 5 years. The treatment exposure in this cohort was 180.4 patient years (Supplementary Figure 3B (12)). During the study period, 3 patients died and 10 other patients discontinued Triac therapy (in 4 cases the parents decided to discontinue due to a lack of perceived benefit; 2 patients discontinued due to financial constraints or unavailability of Triac, and 4 patients discontinued for unknown reasons). Follow-up of the remaining patients continued to January 1, 2021.

**Table 1.** Cohort characteristics

	Off-label use cohort (n = 67)
Age (years)*	4.6 (0.5-66.8)
Age (groups)*	
<2.5 years	23 (34%)
>2.5-5 years	13 (19%)
>5-10 years	17 (25%)
>10-18 years	10 (15%)
>18 years	4 (6%)
Treatment duration (years)	2.2 (0.2-6.2)
>5 years	12 (18%)
2-5 years	26 (39%)
<2 year	29 (43%)
Sex	
Female	0 (0%)
Male	67 (100%)
Continent of origin	
Europe	45 (67%)
Asia	10 (15%)
Oceania	5 (7%)
North America	4 (6%)
South America	2 (3%)
Africa	1 (1%)
Enrollment	
Continuation after Triac Trial I	27 (40%)
Direct off-label use	40 (60%)
T3 concentration (nmol/L)*	4.58 (1.11)
Body weight-for-age (Z-score)*	-2.80 (1.91)
Tachycardia†	21 (35%)

Data are median (range), n (%), or mean (SD).

Abbreviations: T3, triiodothyronine.

*At baseline. Body weight-for-age data were available for 66 patients.

†Tachycardia was defined as a resting heart rate above the 90th percentile for the corresponding age, with cutoffs described by Fleming and colleagues (13). Heart rate data were available for 60 patients (denominator for percentage calculation).

The mean time to reach the maintenance dose was 5.0 (SD 4.7) months, with a mean of 2.7 (SD 1.1) dose increments. The median maintenance dose was 38 µg/kg/day (interquartile range, 31-61 µg/kg/day; range, 15-105 µg/kg/day).

All 67 participants received Triac and had at least 1 follow-up measurement of serum T3 concentrations and thus were included in the analysis of the primary endpoint. Of these 67 patients, 4 patients had not been treated longer than the mean time to optimal dose (5.0 months) and thus 63 participants were included in the analyses of the secondary endpoints. Of these 63 patients, 12 (19%) patients were treated for ≥ 5 years, 10 (16%) patients were treated for 3 to 5 years, 34 (53%) patients were treated for 1 to 3 years, and 7 (13%) patients were treated for < 1 year. Longitudinal within-subject analyses were performed in 38 patients with at least 2 years of follow-up. Timing of the measurement of the different outcomes is shown in Supplementary Figure 4 (12).

**Figure 1.**
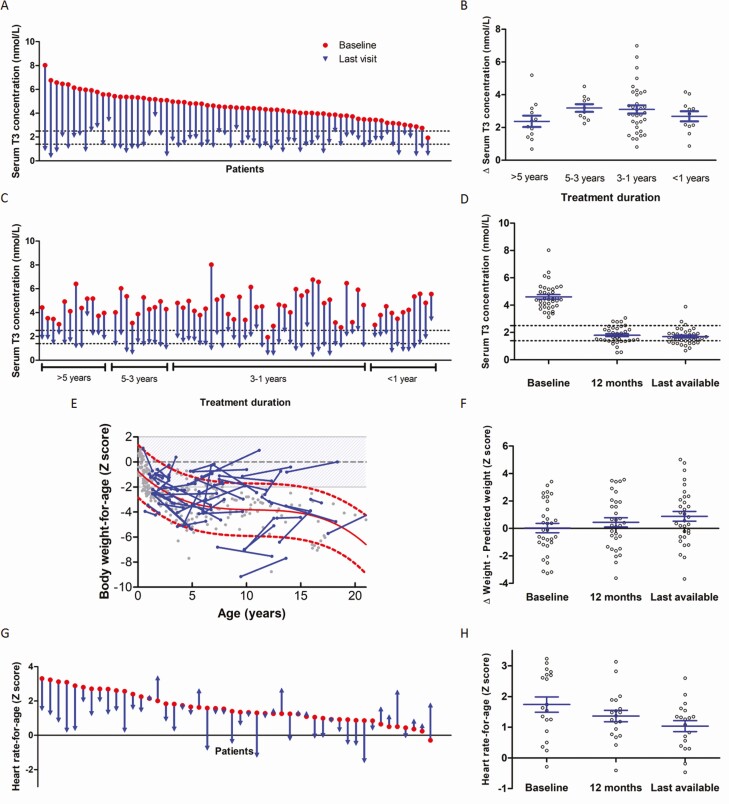
Changes from baseline to interim evaluation and last visit in primary and secondary outcomes (anthropometric parameters). Data are changes in serum concentrations of T3 between baseline and last available follow-up visit on treatment with Triac: A, by patient; B, change in serum T3 between baseline and last available follow-up visit after stratification based on treatment duration; C, by patient, ordered by treatment duration). Panel D shows the change in serum T3 concentrations from baseline to 1 year and last available visit (longitudinal within-subject analyses; n = 36). Panel E shows change in body weight-for-age between baseline and last visit (blue dots and lines; n = 55); the natural history of untreated patients with MCT8 deficiency is depicted in grey dots with the historical reference line in red (with the 95% error band in dashed lines), based on a historical control cohort (8). Panel F shows the change in difference between the body weight-for-age Z-score and the expected Z-score based on the natural history data on the corresponding age from baseline to 1 year and last available visit (longitudinal within-subject analyses; n = 32). Panel G shows change in heart rate-for-age from baseline to last visit (n = 48). Panel H shows the change in heart rate-for-age from baseline to 1 year and last available visit (longitudinal within-subject analyses; n = 19). Median treatment duration was 2.2 years (interquartile range [IQR] 1.2–3.7 years) for analyses of the primary outcome (panels A, B, and C), 2.2 years (IQR 1.5–3.9 years) for analyses of secondary outcomes (panels E and G), and 3.6 years (IQR 2.5–5.2 years) for longitudinal within-subject analyses (panels D, F, and H). Red dots represent baseline measurement and blue arrows represent the last available measurement in panels A, C, and G. Grey dots represent measurements in the individual patients in panels B, D, F, and H; means and standard error of the mean (SEM) are displayed in blue. Black dashed lines represent the target range in panels A, C, and D. Of the patients with a decrease in body weight-for-age compared with the reference line, 7 (78%) of 9 patients were known to have feeding problems, and only 2 (22%) of 9 patients had a feeding tube in place (information not available for 4 patients), compared with 36% of patients in the rest of the group. Body weight-for-age Z scores were calculated using TNO growth calculator and heart rate-for-age Z scores were calculated using the Boston Z score calculator. Abbreviations: T3, triiodothyronine; T4=thyroxine.

In the 67 patients assessed for the primary endpoint, mean serum T3 concentration decreased from 4.58 (SD 1.11) to 1.66 (SD 0.69) nmol/L (mean difference 2.92 nmol/L; 95% CI, 2.61-3.23 nmol/L; *P* < 0.0001; Fig. 1A and Table 2). Sixty (90%) of 67 patients reached T3 concentrations below 2.5 nmol/L, the upper limit of the target range. After stratification for treatment duration, a similar reduction in T3 concentrations was observed across all groups (Fig. 1B and 1C and Supplementary Table 1 (12)). In patients who were treated for more than 2 years, serum T3 concentrations after 1 year of treatment and at the last visit did not significantly differ but were significantly reduced compared with baseline, indicating a sustained treatment effect (Fig. 1D and Supplementary Table 2 (12)).

**Table 2.** Changes from baseline to last visit in predefined outcomes

	Baseline mean (SD)	Last visit mean (SD)	Mean change (95% CI)	*P* value
**Primary outcome**				
T3, nmol/L (n = 67)	4.58 (1.11)	1.66 (0.69)	−2.92 (−3.23 to −2.61)	<0.0001
**Secondary outcomes**				
*Anthropometric parameters and heart rate*				
Body weight, kg (n = 58)	17.8 (12.1)	23.6 (14.5)	5.7 (4.2 to 7.2)	
Weight-for-age Z-score (n = 58)	−2.81 (1.94)	−2.64 (1.81)	0.17 (−0.18 to 0.53)	0.3263
Δ Weight-for-age—predicted weight-for-age Z-score (n = 55)	0.07 (1.83)	0.79 (1.92)	0.72 (0.36 to 1.09)	0.0002
Height, cm (n = 44)	101 (21)	116 (23)	15 (12 to 19)	
Height-for-age Z-score (n = 44)	−1.84 (1.77)	−1.92 (1.51)	−0.09 (−0.50 to 0.32)	0.6705
Δ Height-for-age—predicted height-for-age Z-score (n = 43)	−0.44 (1.38)	0.14 (1.41)	0.58 (0.12 to 1.05)	0.0139
Weight-for-height Z-score (n = 44)	−2.02 (2.49)	−1.50 (2.44)	0.52 (−0.35 to 1.39)	0.2358
Heart rate, bpm (n = 48)	113 (21)	97 (20)	−17 (−24 to −10)	<0.0001
Heart rate-for-age Z-score (n = 48)	1.59 (0.89)	0.96 (1.01)	−0.64 (− 0.98 to −0.29)	0.0005
*Thyroid function tests*				
TSH, mU/L (n = 62)*	3.32 (2.30)	0.95 (0.73)	−2.38 (−2.98 to −1.77)	<0.0001
Free T4, pmol/L (n = 64)	9.5 (2.3)	3.4 (1.6)	−6.1 (−6.7 to −5.4)	<0.0001
T4, nmol/L (n = 63)	54.2 (11.8)	18.1 (9.8)	−36.1 (−39.5 to −32.7)	<0.0001
*Peripheral markers*				
Sex hormone-binding globulin, nmol/L (n = 48)	245 (99)	209 (92)	−36 (−57 to −16)	0.0008
Creatinine, µmol/L (n = 47)	32 (11)	39 (13)	7 (6 to 9)	<0.0001
Creatine kinase, U/L (n = 47)*	110 (87)	128 (80)	18 (−8 to 45)	0.2166

All outcomes were assessed in all patients who received Triac treatment longer than the mean time to optimal dose (5.0 months; N = 64). Data are mean. Body weight-for-age Z-scores were calculated using TNO growth calculator and heart rate-for-age Z-scores were calculated using the Boston Z-score calculator. Abbreviations: bpm, beats per minute; T3, triiodothyronine; T4, thyroxine; TSH, thyroid-stimulating hormone.

*TSH and creatine kinase concentrations were log-transformed to ensure a normal distribution before paired *t* tests were done (nontransformed means [SD] and mean changes [95% CI] are presented for the sake of interpretability).

In the 63 patients analyzed for secondary endpoints, the mean body-weight-for-age Z-score increased, but not statistically significantly, from −2.81 SD (SD 1.94) at baseline to −2.64 SD (SD 1.81) at the last visit (mean difference 0.17 SD; 95% CI, −0.18 to 0.53; *P* = 0.3263), representing a mean increase of 5.7 kg (Table 2). Deterioration of body weight and height with increasing age is part of the natural history of patients with MCT8 deficiency who are not treated with Triac (8). Therefore, the results of anthropometric parameters were expressed against the natural history of untreated patients. The mean body weight-for-age Z-score at baseline was 0.07 SD-points above the natural history reference line and at last visit 0.79 SD-points above the natural history reference line, corresponding to a statistically significant mean increase of 0.72 SD-points (95% CI, 0.36-1.09; *P* = 0.0002, Table 2). Compared with the natural history of treatment-naïve patients with MCT8 deficiency, body weight-for-age improved (> 0.25 SD increase compared with the natural history reference) in 32 (57%) of 55 patients upon treatment and remained stable (−0.25 to 0.25 SD change compared with the natural history reference) in 11 (20%) of 55 patients (Fig. 1E). After stratification for treatment duration, the changes in body-weight-for-age Z-score and body weight-for-age Z-score compared with the natural history reference were similar across all groups (Supplementary Table 1 (12)). In 38 patients who received treatment for more than 2 years, the increase in body weight-for-age from baseline to last visit compared with the natural history of treatment-naïve patients with MCT8 deficiency was more pronounced (mean increase 0.86 SD; 95% CI, 0.31-1.40; *P* = 0.0039; Fig. 1F and Supplementary Table 2 (12)).

The mean body height-for-age increased from baseline to last visit by a mean of 15 cm, although the body height-for-age score did not significantly differ between baseline and last visit (mean difference −0.09 SD, 95% CI, −0.5 to 0.32; *P* = 0.6705) (Table 2). The mean body height-for-age Z-score at baseline was 0.44 SD-points under the natural history reference line and at last visit 0.14 SD-points above the natural history reference line, corresponding with a mean increase of 0.58 SD-points (95% CI, 0.12-1.05; *P* = 0.0139, Table 2). Compared with the natural history of treatment-naïve patients with MCT8 deficiency, body height-for-age improved (> 0.25 SD increase compared to the natural history reference) in 27 (63%) of 43 patients upon treatment and remained stable (−0.25 to 0.25 SD change compared with the natural history reference) in 7 (16%) of 43 patients (Supplementary Figure 5A (12)). After stratification for treatment duration, the changes in body height-for-age Z-score and body height-for-age Z-score compared with the natural history were similar across all groups (Supplementary Table 1 (12)). In 38 patients who received treatment for more than 2 years, the increase in body height-for-age Z-score from baseline to last visit compared with the natural history of treatment-naïve patients with MCT8 deficiency was more pronounced (mean increase 0.89 SD; 95% CI, 0.23-1.55; *P* = 0.0178, Supplementary Figure 5B and Supplementary Table 2 (12)). Weight-for-height Z-scores showed an increasing trend at the last visit compared with the baseline visit, suggesting that the improvement in body weight is not solely driven by linear growth (Table 2). In addition, the effect of Triac on height-for-age development in patients between 8 and 18 years of age was not different in prepubertal vs pubertal patients (Supplementary Figure 5C (12)). The majority of patients between 8 and 18 years show a stabilization or an increase in height-for-age Z-score after treatment, with a subset of patients reaching a (near) normal height-for-age Z-score after treatment. As Z-scores intrinsically account for growth spurts in healthy population, this could imply that Triac treatment enables relatively normal pubertal growth in most patients. Available Tanner stage at the last visit of patients aged from 8 to 18 years during treatment showed delayed sexual maturation in 5 (29%) out of 17 patients (Supplementary Figure 7 (12)), similar to untreated patients with MCT8 deficiency (8). No effects of Triac treatment on sexual maturation were reported in these patients.

Mean heart-rate-for-age Z-score decreased significantly (mean difference −0.64 SD; 95% CI, −0.98 to −0.29; *P* = 0.0005; Fig. 1G and Table 2), improving (> 0.25 SD decrease) in 29 (60%) of 48 patients and remaining stable (−0.25 to 0.25 SD change) in 10 (21%) of 48 patients. The proportion of patients with tachycardia (heart rate above the 90th percentile for the corresponding age, with cutoffs described by Fleming and colleagues (13)) decreased from 18 (38%) of 48 patients at baseline to 11 (23%) of 48 patients at the last visit. After stratification for treatment duration, the change in heart-rate-for-age was similar across all groups (Supplementary Table 1 (12)). In patients who were treated for more than 2 years, a persistent reduction of the mean heart-rate-for-age was observed after 1 year of treatment and at the last visit, with a mean decrease from baseline of −0.38 SD-points after 1 year and −0.70 SD-points at the last visit (Fig. 1H and Supplementary Table 2 (12)).

Mean serum TSH, free T4, and total T4 concentrations significantly declined upon Triac treatment (Table 2). Moreover, after stratification for treatment duration, the change in TSH and total T4 concentrations was similar across all groups (Supplementary Figure 6A and 6E and Supplementary Table 1 (12)). The mean reduction in free T4 concentrations was smaller in patients treated for ≥ 5 years (mean decrease 3.9 pmol/L) in comparison with patients treated for 3 to 5 years (mean decrease 6.6 pmol/L), from 1 to 3 years (mean decrease 6.7 pmol/L) and < 1 year (mean decrease 5.9 pmol/L) (*P* = 0.0055; Supplementary Figure 6C and Supplementary Table 1 (12)). In patients who were treated for more than 2 years, serum TSH, free T4, and total T4 concentrations after 1 year of treatment and at the last visit did not significantly differ but were significantly reduced compared to baseline, indicating a sustained treatment effect (Supplementary Figure 6B, 6D, and 6F and Supplementary Table 2 (12)).

**Figure 2.**
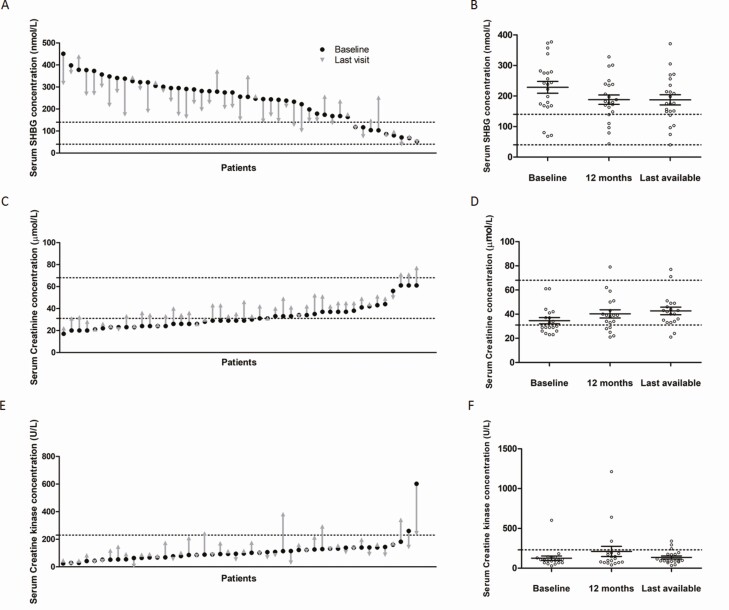
Changes from baseline to interim evaluation and last visit in secondary outcomes (peripheral markers). Panel A shows changes in serum concentrations of SHBG between baseline and last available follow-up visit on treatment with Triac (n = 47). Panel B shows the change in serum SHBG concentrations from baseline to 1 year and last available visit (longitudinal within-subject analyses; n = 22). Panel C shows changes in serum concentrations of creatinine between baseline and last available follow-up visit on treatment with Triac (n = 46). Panel D shows the change in serum creatinine concentrations from baseline to 1 year and last available visit (longitudinal within-subject analyses; n = 19). Panel E shows changes in serum concentrations of CK between baseline and last available follow-up visit on treatment with Triac (n = 46). Panel F shows the change in serum CK concentrations from baseline to 1 year and last available visit (longitudinal within-subject analyses; n = 19; for clarity, data are depicted as nontransformed in panels E and F). Median treatment duration was 2.2 years (interquartile range [IQR] 1.5–3.9 years) for analyses of secondary outcomes (panels A, C, and E) and 3.6 years (IQR 2.5–5.2 years) for longitudinal within-subject analyses (panels B, D, and F). Black dashed lines represent the reference intervals (for the median baseline age). Black dots represent baseline measurement and gray arrows represent the last available measurement in panels A, C, and E. Gray dots represent measurements in the individual patients in panels B, D, and F; means and standard error of the mean are displayed in blue. Abbreviations: CK, creatine kinase; SHBG, sex hormone-binding globulin.

Mean SHBG concentration, reflecting the liver-specific status of thyroid hormone action, decreased from 245 (SD 99) to 209 (SD 92) nmol/L (mean difference 36 nmol/L; 95% CI, 16-57; *P* = 0.0008) (Fig. 2A and Table 2). After stratification for treatment duration, the change in SHBG concentrations was similar across all groups (Supplementary Table 1 (12)). Patients treated for more than 2 years showed a persistent decrease in SHBG concentrations after 1 year of treatment and at the last visit (Fig. 2B and Supplementary Table 2 (12)). Creatinine, reflecting renal thyroid hormone activity, increased from 32 (SD 11) to 39 (SD 13) µmol/L (mean difference 7 µmol/L; 95% CI, 6-9; *P* < 0.0001) (Fig. 2C and Table 2). Patients treated for more than 2 years showed a persistent increase in creatinine concentrations after 1 year of treatment and at the last visit (Fig. 2D and Supplementary Table 2 (12)). In contrast, CK, a marker of thyroid hormone activity in skeletal muscle, showed no statistically significant changes on Triac treatment [110 (SD 87) to 128 (SD 80) U/L; mean increase 18 U/L; 95% CI, −8 to 45; *P* = 0.2166; Fig. 2E and 2F and Table 2].

Complementary analyses of all secondary endpoints on the full analysis dataset (all patients who received at least 1 dose of Triac and had at least 1 follow-up visit after the baseline assessment) showed similar results (Supplementary Table 3 (12)).

No severe adverse events related to Triac were reported during follow-up. Transient signs of increased thyrotoxicosis (increased irritability and anxiety, reduced sleep, increase in blood pressure, and tachycardia) were reported in a small subset of patients (5 of 67 patients; Supplementary Table 4). No hospitalizations related to Triac were reported. During the total treatment period, 3 patients died: a 13-year-old patient died due to sudden death, a 71-year-old patient died after aspiration and a 14-year-old patient died of unknown cause. This is in line with commonly observed causes of death in MCT8 deficiency, with cause of death also often being unknown (8). Postmortem examination was performed in none of the cases. At the time of death, all patients had been on a stable dose of Triac for > 1.5 years, > 4 years, and >2.5 years, respectively.

## Discussion

In this study, we report the long-term (up to 6 years) efficacy and safety of Triac treatment for pediatric and adult patients with MCT8 deficiency. Key clinical and biochemical improvements, documented following 1 year of treatment with Triac, persisted over 6 years of therapy in a real-life clinical setting (11).

The peripheral thyrotoxicosis in MCT8 deficiency is associated with substantial morbidity and, possibly, mortality in this vulnerable population (8). Triac treatment effectively reduced serum T3 concentrations in a rapid and sustainable manner.

Being severely underweight is a key feature of MCT8 deficiency (8). The natural history of body weight- and body height-for-age is nonlinear with a steep deterioration in the first years of life and during puberty (8). Despite more than 50% of the cohort being younger than 5 years of age at baseline and thus prone to a steep decline in body weight- and height-for-age Z-scores, mean age-adjusted Z-scores for both parameters did not further decline on treatment with Triac. Taking into account the natural history data, a substantial proportion of patients exceeded the expected growth. Therefore, the present data indicate that Triac treatment prevents the further deterioration of body weight and linear growth and emphasize the relevance of well-phenotyped historical control cohorts in rare disorders, when a control group is often deemed not feasible for intervention studies. In addition to these anthropometric outcomes, heart rate sustainably improved upon treatment, with the proportion of patients with tachycardia decreased by 40%. In support of the beneficial effects of Triac, biochemical markers reflecting thyroid hormone action in different tissues improved, in line with our previous studies (11). Thus, with being underweight and cardiovascular abnormalities resulting from the peripheral thyrotoxicosis, and with being underweight at young age associated to increased risk for death, amelioration of these key characteristics of MCT8 deficiency by Triac has the potential to diminish morbidity and mortality.

Triac has been administered to different patient populations since the 1950s (14). However, systemic monitoring of side effects has been limited in the past. No adverse effects were reported in hypothyroid patients who were treated for up to 10 months. We previously summarized reports in which the effects of Triac in patients with resistance to thyroid hormone β (RTH-β) were described; 7 out of 34 RTH-β patients were treated for over a year and no adverse effects were reported (15). Triac has also been safely utilized for TSH suppression in patients with differentiated thyroid carcinoma after thyroidectomy with reported data up to 6 months (14). In our Triac Trial I, we reported the occurrence of transient adverse effects likely related to Triac in 6 out of 46 patients with MCT8 deficiency who were treated for 12 months (11). Although enhanced skeletal metabolic activity was observed in athyreotic patients treated with Triac (16), no effects of Triac on bone markers were observed in Triac Trial I. In the present manuscript we provide data on adverse effects during the use of Triac for up to 6 years in the largest cohort study until now. In line with the available data in the literature, the current data support the benign safety profile of Triac treatment in patients with MCT8 deficiency.

The present study expands existing knowledge in different ways. First, it documents that the efficacy of Triac treatment observed in a trial with a limited number of patients, intrinsic to the low prevalence, is preserved in a real-life setting. Post-trial evaluation of therapies can be challenging, particularly for rare disorders. We show that through investigator-initiated joint international efforts, systemic long-term data on a therapy for a rare disorder can be collected in a uniform way. Second, our study confirms that the positive effects on primary and secondary outcomes as reported following 1 year of treatment were sustained up to 6 years of treatment. Third, it demonstrates that the improvements are seen across all age categories. In our previous trial, only 3 patients were younger than 2.5 years, in contrast to 23 patients in our present study. Fourth, our data substantiate the concept that Triac is safe and well-tolerated in the long term.

This study has several limitations. First, it is unknown whether this cohort is fully representative for the entire population of patients with MCT8 deficiency. This cohort, however, represents a significant proportion of known patients (67 patients out of approximately 225 patients known in literature (8)). Second, this observational study collecting data on off-label use of Triac treatment in patients with MCT8 deficiency in a real-life treatment setting provides a less-controlled environment compared with a clinical trial, impeding optimal collection of efficacy and safety data. Patients have potentially also received care outside of their designated center, which could hamper reporting of adverse events. However, with trial patients not necessarily representing patients who get the drug prescribed, real-world data are of great additive value to determine effectiveness for any new therapy, as recently re-emphasized (17, 18). Therefore, the observation that Triac is effective in a real-life setting may further substantiate its beneficial effects. Third, this study precludes determining the optimal Triac dose for MCT8 deficiency for both the peripheral and neurocognitive phenotype as data on neurodevelopmental outcomes had not been uniformly collected. The present international phase IIb clinical trial (NCT02396459) will investigate the effects of Triac on neurodevelopment as well as on the peripheral phenotype in patients with MCT8 deficiency who are younger than 2.5 years.

Taken together, our real-life retrospective cohort study depicts a sustained beneficial effect of Triac on the peripheral phenotype of patients with MCT8 deficiency across all age categories and highlights the potential of Triac as a treatment for MCT8 deficiency.

## Data Availability

Restrictions apply to the availability of some or all data generated or analyzed during this study to preserve patient confidentiality or because they were used under license. The corresponding author will on request detail the restrictions and any conditions under which access to some data may be provided.

## Abbreviations

ANOVA: analysis of variance
CK: creatine kinase
MCT8: monocarboxylate transporter 8
SHBG: sex hormone binding globulin
T3: triiodothyronine
T4: thyroxine
TSH: thyrotropin (thyroid-stimulating hormone)

## Additional Information

***Disclosures:*** The Erasmus Medical Center (Rotterdam, Netherlands), which employs F.S.v.G., S.G., E.L.T.v.d.A., S.A.A.v.d.B., H.v.T., Y.B.d.R., M.d.W., R.v.d.W., R.P.P., and W.E.V. receives royalties from Rare Thyroid Therapeutics (the manufacturer of Triac), dependent on commercialization. None of the authors will benefit personally from any royalties. Rare Thyroid Therapeutics had no influence on the conduct or analysis of this study. All other authors declare no competing interests.
